# Children and Malaria

**Published:** 1900-03-31

**Authors:** 


### CHILDREN AND MALARIA.

The facts elicited by the researches of Professor Koch in Java and by Professor Celli in the Roman Campagna in regard to the incidence of malaria upon children are of special importance as tending to explain the terribly high infant mortality in malarious districts and the comparative immunity to the disease in later life which is enjoyed by some of those who are born and brought up in malarious districts. Koch points out that the tendency to malaria in a district is best measured by the frequency of its occurrence in children as shown by blood examination, for by virtue of having had attacks in infancy the adults may have attained a more or less complete immunity, and may thus give no evidence of the malarious character of the place. By examining the blood of a large number of children he was able to show how exactly the tendency to malaria among them runs parallel with the abundance of anopheles, and with the existence of ponds suitable for their breeding. In the same way also he was able to explain some of the apparently anomalous cases in which malaria was found to exist in districts which seemed to be free from the mosquitos usually associated with it, for in these places it was found that the disease was confined to adults who had had opportunities for contracting the infection in the plains below, while the examination of the blood of a large number of the children showed a complete absence of the parasite. It would seem then that to ascertain whether or no a given district is malarious one must examine the blood of the children rather than that of the adults, for the latter may on the one hand have become immune in consequence of having been soaked with the disease in infancy, and on the other, although they may suffer from the disease, they may have caught it in some quite different locality. These researches go far to prove that the high infant mortality of the tropics is largely a matter of malaria; and to exp ain how it is that the children of non-immune persons, such as newly arrived Europeans, are placed at such serious disadvantage as is notoriously the case in tropical countries.

